# Supplementary material for: Ultrasensitive imaging-based sensor unlocked by differential guided-mode resonance
Source: Nat Commun. 2025 Jul 3;16:6113. doi: 10.1038/s41467-025-60947-3 (PMC12229338; doi:10.1038/s41467-025-60947-3)
Supplement: Supplementary file 1 — Supplementary Information [file 41467_2025_60947_MOESM1_ESM.pdf]

Supporting Information

Ultrasensitive Imaging-based Sensor Unlocked by Differential Guided-Mode Resonance

Zhenchao Liu[1, 2, 3#], *Houxin Fan*[1#], *Tingbiao Guo*[1]*, *Qin Tan*[1], *Zhi Zhang*[1], *Yuwei Sun*[1], *Julian Evans*[1], *Junbo Liang*[4], *Ruili Zhang*[4] and *Sailing He*[1, 2, 4, 5]*

[1] Centre for Optical and Electromagnetic Research, Enze-ZJU Joint Lab for MedEngInfo Collaborative Innovation, College of Optical Science and Engineering, Zhejiang University (ZJU), Hangzhou, 310058, People's Republic of China

[2] Taizhou Institute of Medical Health and New Drug Clinical Research, Taizhou Enze Medical Center (Enze), Taizhou Hospital, Zhejiang University, Taizhou, 318000, People's Republic of China

[3] Singapore University of Technology and Design (SUTD), 8 Somapah Road, Singapore, 487372, Republic of Singapore

[4] National Engineering Research Center for Optical Instruments, Zhejiang University, Hangzhou, 310058, People's Republic of China

[5] Department of Electromagnetic Engineering, School of Electrical Engineering, KTH Royal Institute of Technology, Stockholm, SE-100 44, Sweden

#These authors contributed equally to this work.

*Corresponding authors: sailing@kth.se and tbguo@zju.edu.cn

Contents

1. Methods 3

1.1 Numerical Simulation 3

1.2 Design and fabrication 3

1.3 Measurement and characterization 4

1.4 The Transfer Matrix Method (TMM) calculation 5

2. The localized electric field distribution on the surface of the sensing structure 6

3. The measured refractive index (RI) of SU8 epoxy resin after exposure curing 7

4. The thickness information of four dielectric patches 8

5. The Q-factor of the resonance 10

6. The exposure dose matrix 11

7. The relationship between refractive index and concentration of glucose solution 12

8. The angle scanning and thickness extraction 13

9. The extraction of the refractive index 15

9.1 Building the "binary image library" for different refractive indices 15

9.2 Image matching process 15

10. The expandable dynamic range (Δn) and the applicable area of RI ((n_1, n_2)) 19

11. The relationship between sensitivity, range, resolution and thickness gradient 21

11.1 The sensitivity-gradient relationship 21

11.2 The range-gradient relationship 23

11.3 The refractive index resolution 25

12. The fineness of the stripe and the calculation of the Figure of Merit (FOM) 27

13. The repeated measurement error 28

14. The method to determine the pixel shift of the ring stripe 29

15. The detailed modification and some results regarding the biotin test in Figure 5a 30

16. The two-dimensional dynamic perception for refractive index or particle distribution 32

17. The details on the sensitivity of the imaging sensor to variations in the incident angle and the collimation of the incident waves 35

18. The comparison between image-based refractive index sensors and other types of refractive index sensors 37

19. Detailed explanation of dGMR and the physical mechanism behind high sensitivity 38

20. The repeatability assessment for the sensor chip fabrication based on PECVD 39

21. The detailed fabrication process for the sensor chip. 41

22. The thickness controlling experiment through EBL dose-modulation patterning. 42

References 43

1. Methods

1.1 Numerical Simulation

The transfer matrix method (TMM) calculation for multi-layer resonant structure at 10 nanometer scale is simulated by MATLAB. For Figure 2 and Figure 3, the waveguide layer is made up of the SU-8 1030 photoresist. The refractive index of the SU-8 photoresist is determined by measurement. The refractive index of other materials is taken from the refractive index library. Other simulation details of Figure 2b(i) and (ii) are: the wavelength of incident lightwave (λ=671 nm), 1st layer (prism layer/substrate layer, ε_1=2.3043), 2nd layer (titanium adhesive layer, ε_2=-7.2269+i21.580), 3rd layer (silver layer, ε_3=-20.917+i0.43400), 4th layer (SU-8 waveguide layer, n=1.5995, t=450 nm), 5th layer (sensing medium layer, n ranges from 1 to 1.45 in Figure 2b(i), n=1.33 in Figure 2b(ii)). In Figure 4, the waveguide layer is made up of silica. The refractive index of silica is determined by measurement. The construction of the "binary image library" and image matching process are achieved by the homemade MATLAB scripts. The guided wave model in Figure 2 is calculated through MATLAB, based on the phase matching condition. The localized electric field distribution is calculated using FDTD software. The materials parameters are adopted from the built-in materials library. The recovery of the thickness profile in Figures 2-4 is achieved with homemade MATLAB scripts based on the TMM model.

1.2 Design and fabrication

For Figures 2 and 3, the fabrication process of the four-patch chip and the code-like sensing chip includes photolithography, metal evaporation, lift-off process, electron beam lithography and microfluidic chip sealing. The photolithography process includes spin coating, pre-baking, exposure, and development. AZ5214 photoresist is used for lithography with a mask aligner machine (SUSS Micro Tec, MA6). Titanium and silver are evaporated (Denton, Explorer) with thicknesses of approximately 2 nm and 50 nm, respectively, supporting the SPP mode. After that, the lift-off process is carried out, using acetone and ultrasound to remove the photoresist. In the electron beam exposure process (Raith 150 TWO), we use SU-8 1030 as the electron beam resin layer, whose thickness can be affected by the exposure dose. The microfluidic chip sealing is achieved with the plasma cleaner (Harrick, PDC-002) through the covalent bonding method. In Figure 3, the "QR" patches (waveguide layer) with different thicknesses are

produced by controlling the exposure dose in the electron beam exposure process. In Figure 4, the fabrication process for patterning the Ag layer to make a sensor array on a 4-inch wafer includes photolithography, metal evaporation, lift-off process, silica deposition and microfluidic chip sealing. The silica is deposited by the PECVD (made by Surface Technology Systems Ltd., model M/PLEX CVD), with the thickness difference introduced through the deposition error. The multi-channel sensing chip is achieved through the array-type microfluidic chip technology based on the silicon substrate mold fabricated by the lithography process. The optic mounts of the prototype in Figure 5 are designed and fabricated with customized 3D printing technology. The humidity sensing box in Figure 5 is based on a homemade air chamber, which is built using an acrylic board.

1.3 Measurement and characterization

In Figures 2 and 3, for the angle scanning process we use a programmable rotation platform. The rotation platform rotates step-by-step while the reflective images are captured by a CMOS camera. The pair of prisms make the direction of reflective light nearly unchanged during the angle scanning process. The optical microscope images in Figures 2 and 3 are obtained with the Olympus BX53M microscope. For the standard thickness measurement of the four patches in Figure 2, we use the thickness meter (Filmetrics F40-UV). The QR code in Figure 3 is generated using the QR code generation website. We scan the QR code in Figure 3 with a smartphone. The light source used in this system is a 671 nm laser (MRL-III-671-100mW). The characterization of the refractive index of the SU-8 epoxy resin and silica layer is achieved by an ellipsometer (HORIBA, UVISEL). In Figure 3, solutions with different concentrations (from 0% to 10%) are sequentially injected into the microfluidic channel and come into contact with the surface of the sensing chip. After each injection process, the reflected images are captured by a CMOS camera. In Figure 3, the polydopamine (PDA) tris solution (pH=8.5) is injected into the microfluidic channel, with the reflected image recorded every five minutes to monitor the molecule absorption process on the surface of the sensing chip. In Figure 4, the angle scanning process is achieved through a programmable rotation platform with a small step. The thickness profile of the silica waveguide layer is fitted based on the TMM model. In Figure 4, solutions with different concentrations (from 0.0% to 1.0%) are sequentially injected into the microfluidic channel and come into contact with the surface of the sensing chip. After each

injection, the reflected images are captured through a CMOS camera to record the position of the ring stripe. In the multi-channel sensing test in Figure 4, the solutions with different concentrations are injected into different channels. The humidity testing in Figure 5 is performed in an air chamber. The change in humidity is produced by a humidifier. During this testing, the standard humidity is characterized at the same time with a commercial hygrometer (CEM, DT-625).

1.4 The Transfer Matrix Method (TMM) calculation

The transfer matrix of the multi-layer system can be expressed as:

$$M^{(i)} = \begin{pmatrix} M_{(1,1)}^{(i)} & M_{(1,2)}^{(i)} \\ M_{(2,1)}^{(i)} & M_{(2,2)}^{(i)} \end{pmatrix} = \begin{pmatrix} \cos\left(p^{(i)}\right) & -i\frac{\sin\left(p^{(i)}\right)}{q^{(i)}} \\ -iq^{(i)}\sin\left(p^{(i)}\right) & \cos\left(p^{(i)}\right) \end{pmatrix} \tag{S1}$$

Here, $p^{(i)} = k_0 t^{(i)}\sqrt{{n^{(i)}}^2 - {n^{(1)}}^2\sin\theta}$ and $q^{(i)} = \frac{1}{{n^{(i)}}^2}\sqrt{{n^{(i)}}^2 - {n^{(1)}}^2\sin\theta}$. $k_0 = \frac{2\pi}{\lambda}$ means the wave vector in vacuum. $t^{(i)}$ is the thickness of ith layer. $n^{(1)}$ and $n^{(i)}$ are the complex refractive indexes of first and ith layer, respectively. θ is the incident angle of the beam. The total transfer matrix can be calculated as:

$$M_{total} = \prod_i M^{(i)} \tag{S2}$$

The complex reflection coefficient is:

$$\frac{q^{(1)}\left(M_{total\,(1,1)} + q^{(i)}M_{total\,(1,1)}\right) - M_{total\,(2,1)} + q^{(i)}M_{total\,(2,2)}}{q^{(1)}\left(M_{total\,(1,1)} + q^{(i)}M_{total\,(1,1)}\right) + M_{total\,(2,1)} + q^{(i)}M_{total\,(2,2)}} \tag{S3}$$

where, $r = |\tilde{r}|$ is the total reflection coefficient of multi-layer films.

2. The localized electric field distribution on the surface of the sensing structure

The external electric field distribution improves the sensing performance for the surrounding medium[1,2]. Due to the coupling between the SPPs (surface plasmon polaritons) and guided-mode resonance, the electric field is localized near the outside of the waveguide layer. Based on the Finite-Difference-Time-Domain (FDTD) method, the electric field of our resonant structure can be calculated, as shown in Figure S1, indicating the electric field is located near the outside of the waveguide layer, and the electric field distribution area is only a few hundred nanometers, indicating its surface sensing capability.

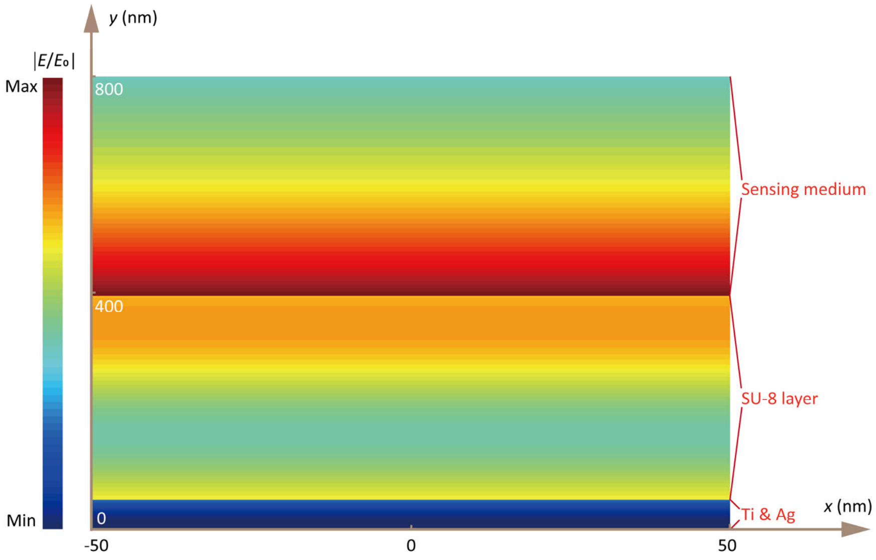

Figure S1. The localized external electric field distribution on the surface in Figure 2a. $|E/E_0|$ in this figure stands for the enhancement factor of the electric field.

3. The measured refractive index (RI) of SU8 epoxy resin after exposure curing

We measured the refractive index of SU8 epoxy resin (GM 1030) with the ellipsometer (HORIBA, UVISEL). The curves for the real and imaginary parts of the measured refractive index are shown in Figure S2, with the measured wavelength ranging from 245 nm to 2100 nm. The real part of RI is 1.5995 (at 671.0 nm), and the imaginary part of RI is 0.0000 (at 671.0 nm). The measured thickness is 453.90±5.04 nm.

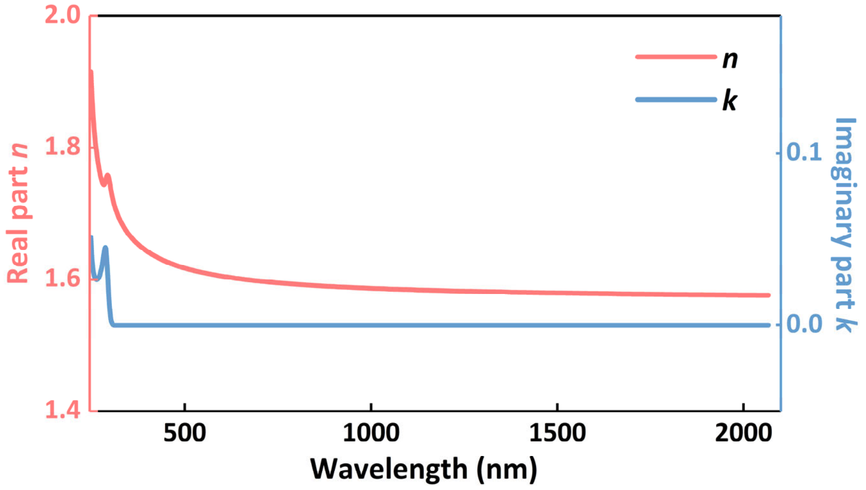

Figure S2. The measured refractive index (RI) of SU8 epoxy resin after exposure.

4. The thickness information of four dielectric patches

We created four-square patterns using epoxy resin (SU-8), silver, titanium, and a K9 glass substrate to demonstrate how the adjusted thickness tunes the resonant angle. The SU-8 epoxy resin is sensitive to exposure dose and allows for easy thickness adjustment[3]. The construction of the testing system is shown in Figure S3. After obtaining the angular spectrum, the resin layer's thickness can be extracted using the TMM model. The comparison between the thickness measured with a thickness meter (Filmetrics F40-UV) and the extracted thickness is shown in Figure S4, indicating high accuracy of the present angle scanning method for extracting the thickness in a small range of 15 nm (435 nm to 450 nm). With the thickness information obtainable through this angle scanning method, the thickness-modulated dielectric layer can serve as an indicator of refractive index changes, as varying the refractive index will excite resonant modes in the dielectric layer with different thicknesses.

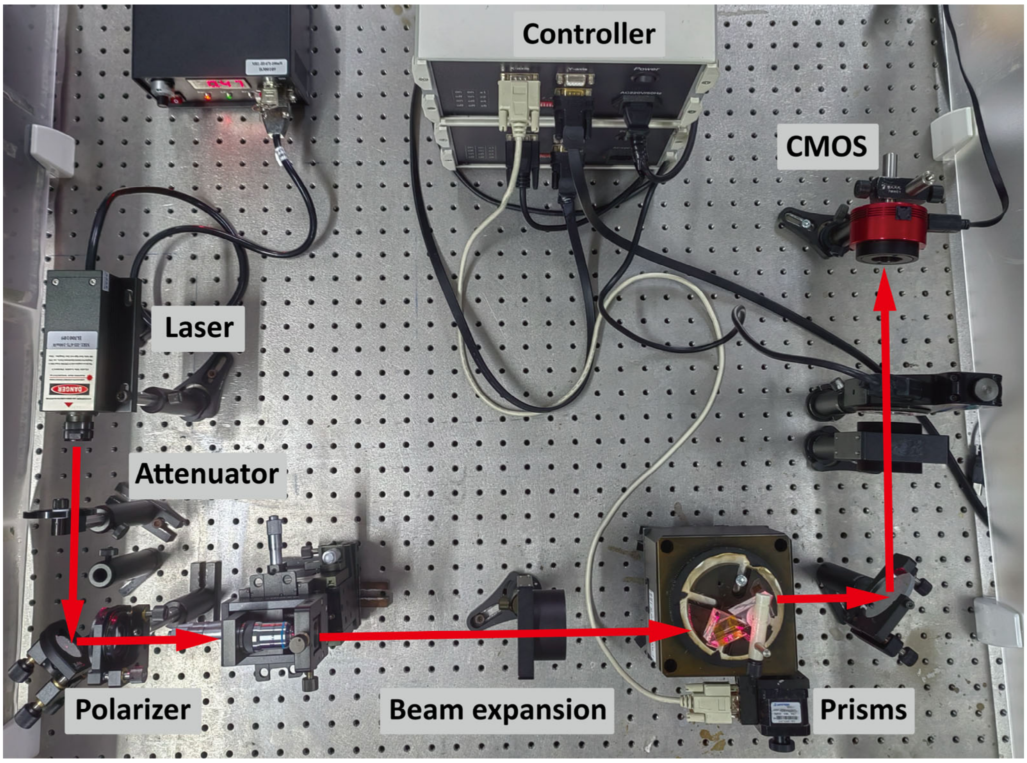

Figure S3. The picture of the optical testing system. The red arrows indicate the light trace of the optical testing system.

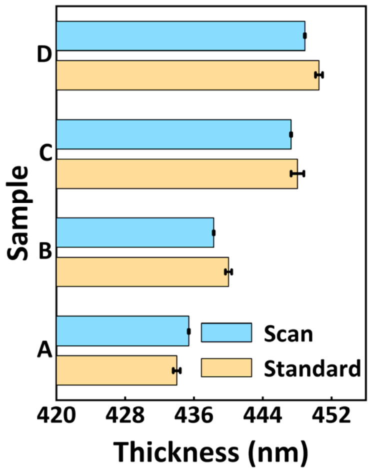

Figure S4. The comparison between the thickness ("Standard") measured with a thickness meter (Filmetrics F40-UV) and the extracted thickness ("Scan"). The characters "A", "B", "C" and "D" represent Samples A, B, C and D in Figure 2f.

5. The Q-factor of the resonance

In Figure 2e, the increase in the Q factor is constrained by the coupling conditions. To elucidate this, we propose a simple physical model based on the phase matching condition of the guide wave, referred to as the guide wave model shown in Figure 2e. The phase matching condition is expressed as:

$$\frac{2\beta t\cos(\theta_d)}{\sin(\theta_d)} - \varphi_d - \varphi_m = 2\pi \tag{S4}$$

Here, β is the propagation constant of the surface wave on the metal-dielectric interface, t is the thickness of the dielectric layer, θ_d is the generalized incident angle in the dielectric layer, φ_d is the phase delay from the dielectric, and φ_m is the phase delay from the metal layer

$$\begin{cases} \theta_d = \sin^{-1}\left(\dfrac{n_p \sin(\theta_{in})}{n_d}\right) \\ \varphi_d = \tan^{-1}\left(\left(\dfrac{n_d}{n_s}\right)^2 \dfrac{(n_d{}^2\sin^2(\theta_d) - n_s{}^2)^{\frac{1}{2}}}{\cos(\theta_d)}\right) \\ \varphi_m = \tan^{-1}\left(2n_d n_m{}^2\cos(\theta_d)\dfrac{\left(\dfrac{-2k_m}{n_m}u - \left(1-\dfrac{k_m{}^2}{n_m{}^2}\right)v\right)}{n_m{}^4\left(1+\dfrac{k_m{}^2}{n_m{}^2}\right)^2\cos^2(\theta_d) - n_d{}^2(u^2+v^2)}\right) + \dfrac{\pi}{2} \end{cases} \tag{S5}$$

Here, n_d, n_p, n_m and n_s are the real part of the refractive index of the dielectric layer, prism layer, metal layer and sensing layer, respectively. θ_{in} is the incident angle in the prism layer. k_m is the imaginary part of the refractive index of the metal layer. In Eq. S5, u and v are the substituted parameters, which can be calculated by (through the generalized Snell's law)

$$\begin{cases} u = \mathrm{Re}\left((n_m + \mathrm{i}k_m)\left(1-\left(\dfrac{n_d\sin(\theta_d)}{n_m+\mathrm{i}k_m}\right)^2\right)^{\frac{1}{2}}\right) \\ v = -\mathrm{Im}\left((n_m + \mathrm{i}k_m)\left(1-\left(\dfrac{n_d\sin(\theta_d)}{n_m+\mathrm{i}k_m}\right)^2\right)^{\frac{1}{2}}\right) \end{cases} \tag{S6}$$

The relationship between the thickness t and incident angle θ_{in} in Figure 2e demonstrates the close alignment of the guide wave model with the rigorous TMM model.

6. The exposure dose matrix

By controlling the exposure dose of electron beam lithography (EBL, Raith 150 TWO), we can control the final thickness of the SU-8 resin layer. The exposure dose matrix for all pixels (or all patches; see Figure S5) is imported into the software of the EBL machine for gray-scale lithography.

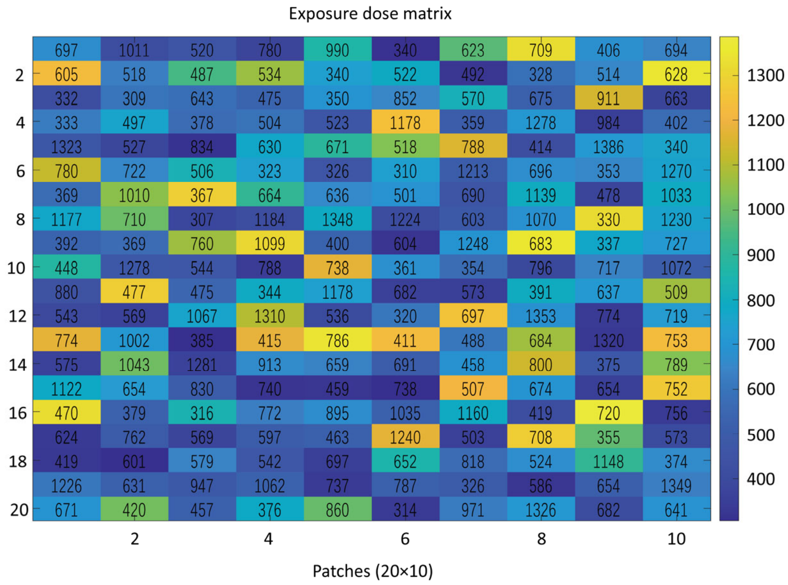

Figure S5. The exposure dose matrix for all pixels in Figures 3c and 3d. The number in each square is the exposure dose for each patch during the electron beam lithography.

7. The relationship between refractive index and concentration of glucose solution

Here, we used the Abbe refractometer (2WAJ) to measure the real refractive index of the glucose solutions with different concentrations (wt%). The relationship between the refractive index and concentration of glucose solution is shown in Figure S6.

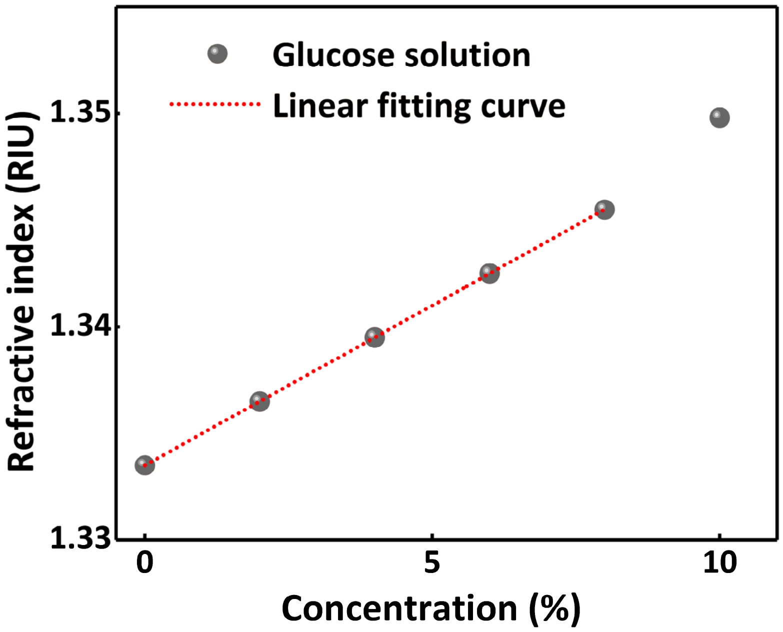

Figure S6. Relationship between refractive index and concentration of glucose via Abbe refractometer.

Figure S6 shows the near-linear relationship between the refractive index and concentration. The RI of the solution with the largest concentration deviated slightly from the linear fit, while the smaller concentration solutions maintain linearity, which is also reported in other literature[4,5]. In the subsequent experiments, we use the solutions within the small concentration range, so linearity can be approximated. The linear equation is:

$$n = 0.0015 \times c(\%) + 1.3335 \tag{S7}$$

Here, n means the refractive index, c (%) means the concentration percentage. The R-square of the linear fitting is 1 in Eq. S7. The measured refractive index from the Abbe refractometer is n_D (at 589.3nm). The correction of the refractive index is required as we use the light source at 671 nm. The correction is according to the data and dispersion formula from S. Kedenburg[6]. Assuming that the solution and water at low concentrations satisfy an approximately equal dispersion relationship. The corrected linear equation is:

$$n = 0.0015 \times c(\%) + 1.3311 \tag{S8}$$

We can infer that if concentration changed 0.1%, refractive index would change 0.00015 RIU.

8. The angle scanning and thickness extraction

The electrical rotation platform is programmed to rotate step by step, enabling scanning of the incident angle. The pair of prisms, composed of two centrally symmetrical prisms, keep the reflected light from the prisms in nearly the same direction while adjusting the incident angle, which improves image capturing. After the angle scanning, the resonance reflective angular spectrum of each pixel (i.e. each patch) in Figure 3d can be obtained. The angular spectrum is scanned and measured under the condition of n=1 (air) and processed to be normalized. As shown in Figure S7, we present some angular spectra of these patches, showing the effect of exposure dose on thickness (and then on the angular spectrum).

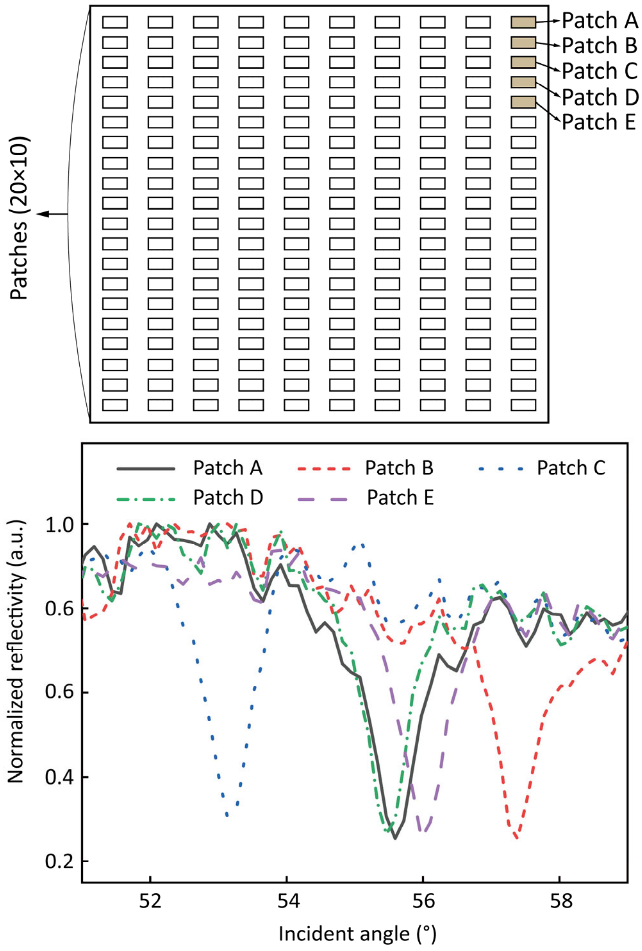

Figure S7. The resonance reflective angular spectrum of some pixels (i.e. patches) in Figure 3d.

We can obtain the resonance angle of each patch, according to the angular spectrum, as shown in Figure S8. Based on the TMM model, there is a one-to-one correspondence between the resonance angle and the thickness of the waveguide layer, which is also depicted in Figure

2b(ii). Then we can obtain the thickness of the waveguide layer of each patch, as shown in Figure S9.

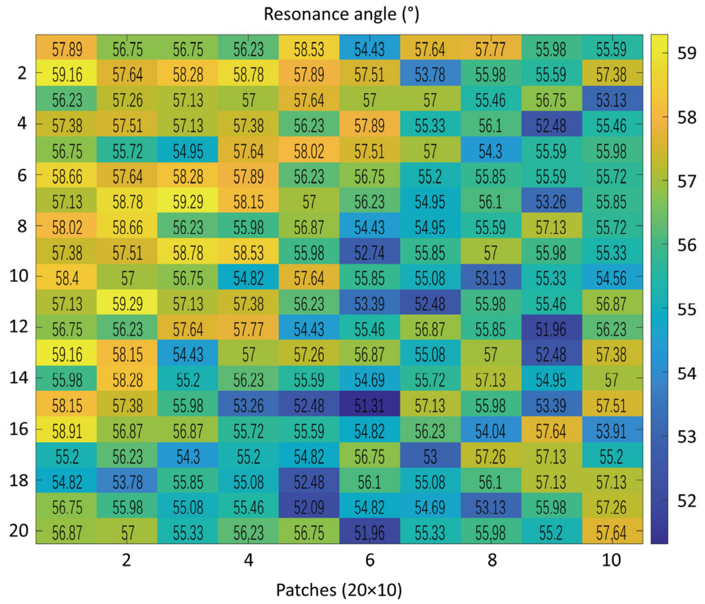

Figure S8. The resonance angle of each pixel in Figure 3d.

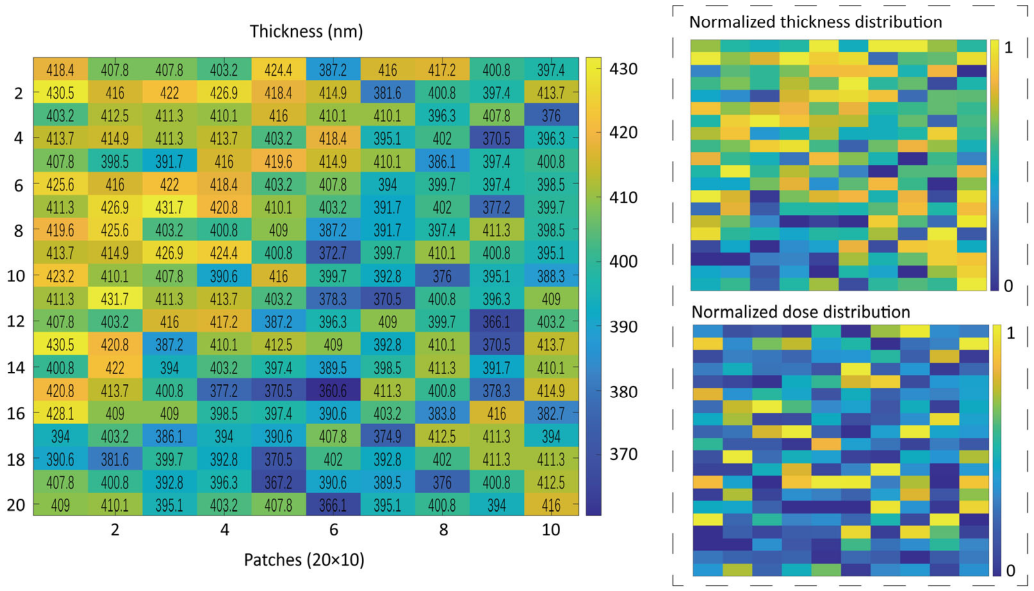

Figure S9. The calculated thickness of each pixel in Figure 3d and the comparison between the thickness distribution and exposure dose distribution.

9. The extraction of the refractive index

The process is divided into two parts, namely, the establishment of the "binary image library" for different refractive indices and the image matching process.

9.1 Building the "binary image library" for different refractive indices

To build the "binary image library" for different refractive indices, one needs to obtain the thickness for each SU8 patch in the sensing chip first. For this, we captured a series of images by incident angle scanning. These captured images were denoised by a Wiener filter. Then affine transformation was used to reshape them to standard rectangles. We then cropped these images to the same size (yellow box in Figure 3).

20×10 rectangle pixel windows were adopted to extract the average intensity values for each patch in the array (blue box in Figure 3). The angular spectrum, especially the resonant angle for each patch could be obtained using these average intensity values. The thickness for each patch was then determined by one-to-one mapping between the thickness and resonant angle. With the thickness profile, the "binary image library" for different refractive indices could be built based on the TMM model.

9.2 Image matching process

To simplify the matching process, we binarize both the experimentally captured images and the simulated images with specific thresholds. The image matching process is shown in Figure S10.

For images in Figure 3, we binarized the experimental images with a threshold of 0.27. We assign the average intensity values larger than 0.27 as '1' and below 1 as '0' (gray box in Figure 3).

For images in Figure 4, we binarized the experimental images with a threshold of 0.5. We assign the average intensity values larger than 0.5 as '1' and below 1 as '0'.

To obtain the refractive index, the structural similarity index measure (SSIM) between these experimental images and simulated images in the "binary image library" for different refractive indices was calculated. The SSIM in MATLAB is defined as

$$SSIM(x,y) = \frac{(2\mu_x\mu_y + C_1)(2\sigma_{xy} + C_2)}{(\mu_x^2 + \mu_y^2 + C_1)(\sigma_x^2 + \sigma_y^2 + C_2)}$$

where μ_x, μ_y, σ_x, σ_y, σ_{xy} are the *x* and *y* mean value, standard deviation and covariance. C_1, C_1 and C_3 are the regularization constants.

The refractive index was finally determined by finding the maximum SSIM value.

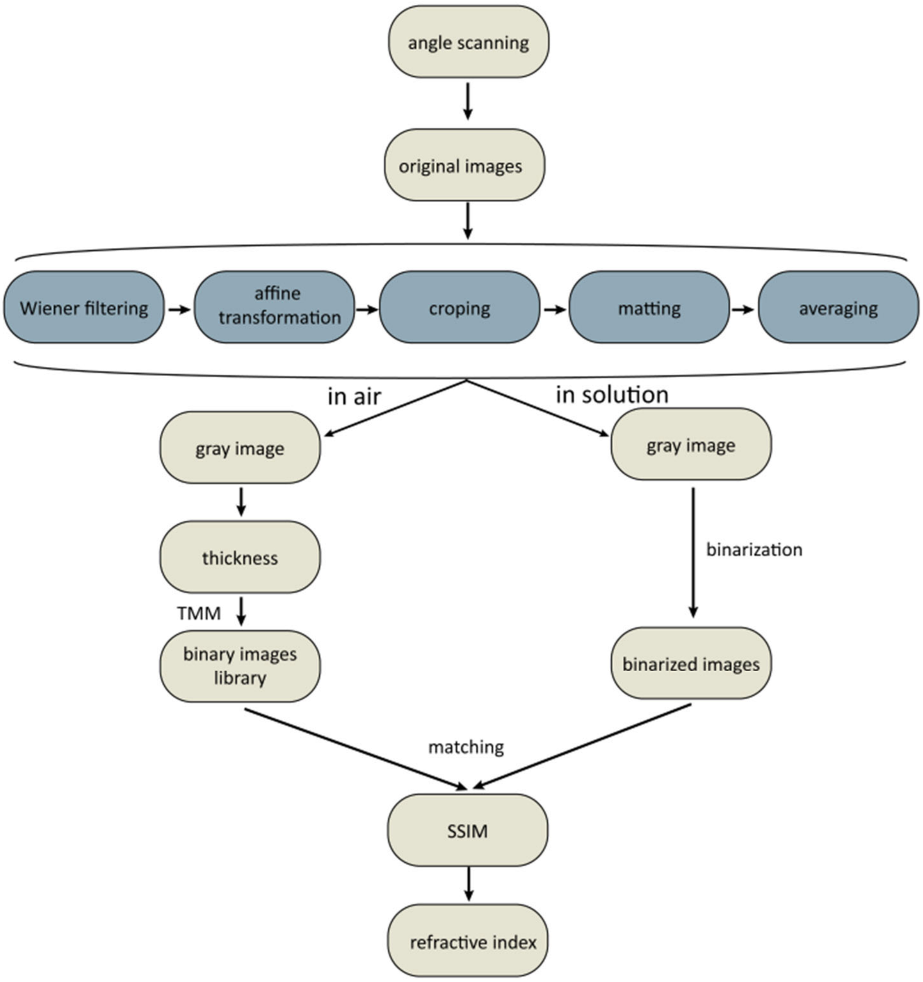

Figure S10. Image matching process algorithm.

The original captured images of different concentrations are shown in Figure S11. Figure S12 shows the pixelated gray images with the extraction of gray value (through filtering and averaging, etc.) from the original captured images. Then the gray images are binarized to binary images, as shown in Figure S13.

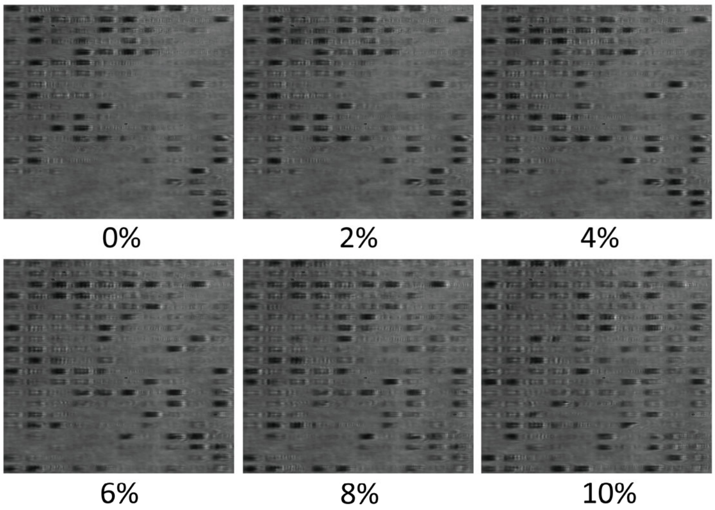

Figure S11. The original captured images of different concentrations.

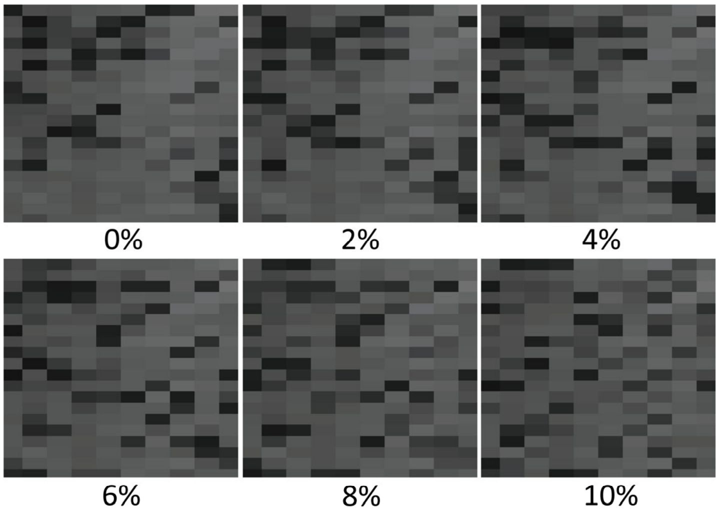

Figure S12. The gray images of different concentrations extracted from captured images.

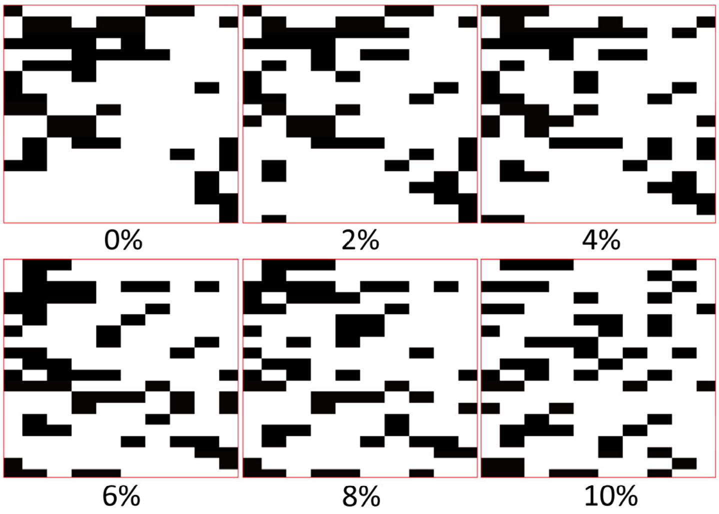

Figure S13. The binary images of different concentrations binarized from gray images.

After obtaining the binary images, the comparison between the measured binary images and the calculated “binary images library” of different refractive indices (or different concentrations) is implemented in MATLAB. Figure S14 shows the SSIM index results for each concentration.

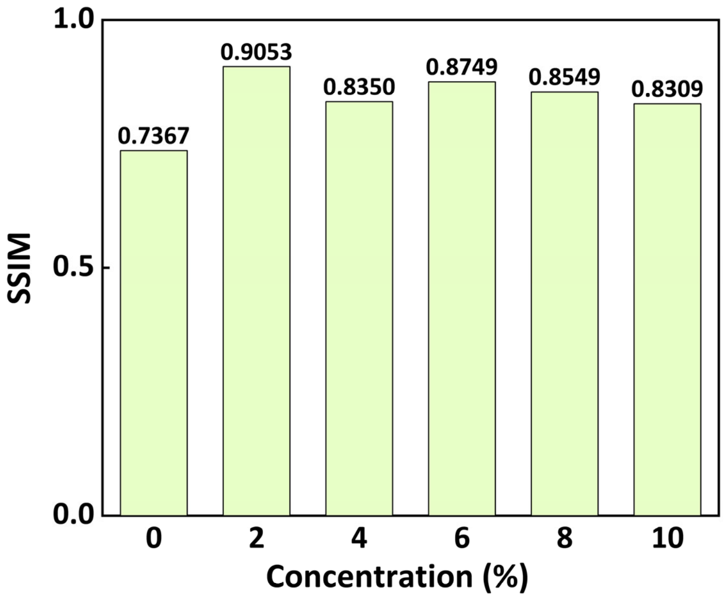

Figure S14. The structural similarity index (SSIM) results for each concentration for the comparison.

10. The expandable dynamic range (Δn) and the applicable area of RI ((n_1, n_2))

Based on the theoretical analysis of Figure 2, we can establish the physical model for the thickness-modulation sensing structure to analyze the theoretical expandable dynamic range and the applicable area of RI expressed as Δn and (n_1, n_2), respectively. The profile of the surface (or the thickness of the dielectric layer) can be assumed to smoothly range from 462 nm to 490 nm among the 20 mm (diameter) chip size, based on the measured profile of Figure 4a. As shown in Figure S15, according to the relationship between the reflectivity and thickness, when n=1 and the incident angle is 53.7220°, the stripe just appears on the edge position where the waveguide thickness is 490 nm. Therefore, the lower limit of the RI is 1 (n_1=1). As the right part of Figure S15 shows, when n=1.3675 and the incident angle is 64.2705°, the stripe just appears on the edge position where the waveguide thickness is 490 nm. Owing to the cut-off phenomenon in Figure 2e, the higher RI will make the stripe leave the edge position of the chip no matter how we adjust the incident angle. Therefore, the upper limit of RI is 1.3675 (n_2=1.3675). The expandable dynamic range Δn= n_2-n_1=0.3675 RIU. The applicable area of RI is (1, 1.3675). Moreover, if we increase the total thickness while keeping the gradient of thickness unchanged, owing to the change of the cut-off condition, the upper limit of RI (n_2) will increase until it is close to the refractive index of the dielectric layer.

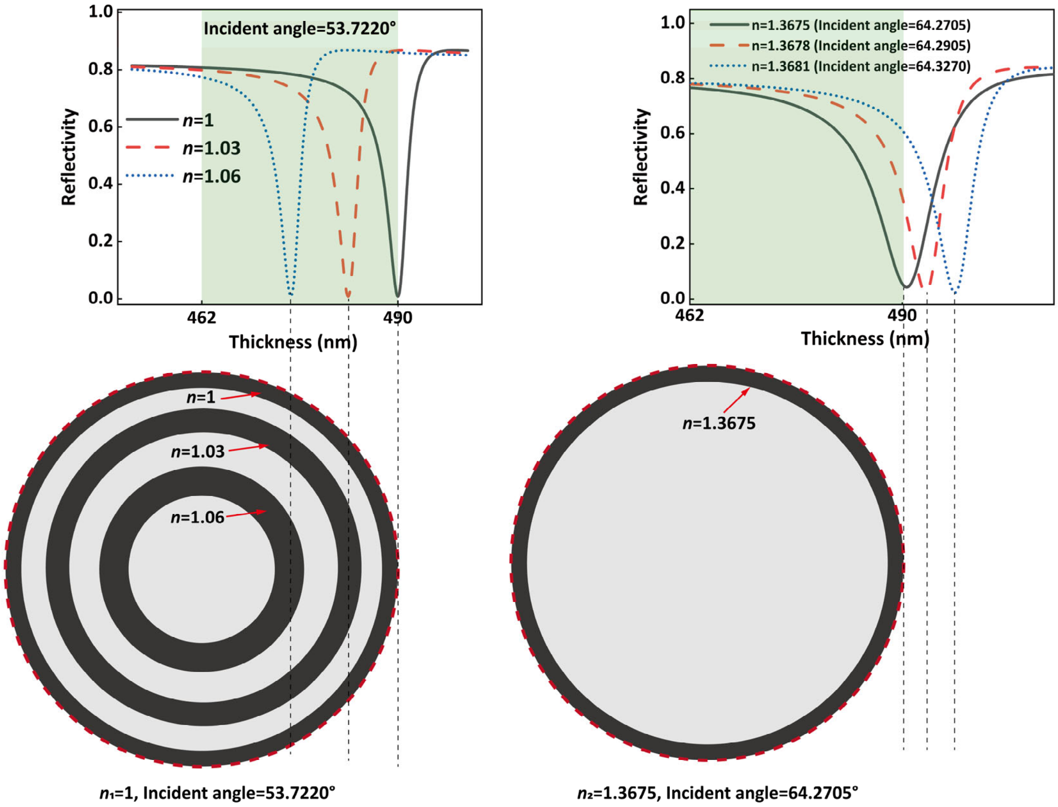

Figure S15. The expandable dynamic range and the applicable area of RI based on the angle scanning. The green area in the figure means the thickness range of the chip (from 462 nm to 490 nm).

11. The relationship between sensitivity, range, resolution and thickness gradient

The calculation details of the sensitivity-gradient relationship, range-gradient relationship and the refractive index resolution are discussed here.

11.1 The sensitivity-gradient relationship

S is the sensitivity (i.e., the shift pixels per unit refractive index change), which is:

$$S = \frac{2(P_2 - P_1)}{n_2 - n_1} \tag{S9}$$

Here, P_1 and P_2 are the position of the ring stripe pattern (i.e. the radius of the ring stripe) under different refractive index conditions (n_1 and n_2), respectively. Since we measure the pixel shift based on the ring's diameter rather than its radius, this introduces a two-fold relationship in the formula above. The position of the ring stripe is determined by the relative position of this area on the sensing chip, which means:

$$S = \frac{2C_1({P_2}' - {P_1}')}{n_2 - n_1} \tag{S10}$$

Here, ${P_1}'$ and ${P_2}'$ are the position of the stripe area on the sensing chip. C_1 means a scale coefficient. The spatial changing rate (t_R) of the thickness t, regarding these two positions can be described as:

$$\begin{cases} t_R = \dfrac{t_2 - t_1}{{P_2}' - {P_1}'} \\ \nabla t = \lim\limits_{{P_2}' \to {P_1}'} \dfrac{t_2 - t_1}{{P_2}' - {P_1}'} = \dfrac{dt}{dx} \end{cases} \tag{S11}$$

Furthermore, dGMR refers to differential of the guided-mode resonance, where the critical component is the differential nature, representing the difference between the two resonances. In this model, we use the height/thickness differences to create the dGMR mode (introducing the "d" in dGMR), which allows us to map changes in refractive index (i.e., the resonance condition of the GMR) to spatial changes in the resonance stripes. Different stripes have different heights/thicknesses of the GMR mode. This mapping reveals a novel mechanism for sensitivity enhancement, which is why we need to incorporate the height/thickness parameters related to spatial stripe variation into the sensitivity calculation formula. According to the definition of the thickness gradient ∇t, when ${P_2}'$ tends towards ${P_1}'$, t_R stands for the thickness gradient ∇t, only considering one dimension. Therefore, the product of ∇t and S can be expressed as:

$$\nabla t \cdot S = 2C_1 \frac{dt}{dn} \tag{S12}$$

The relationship curve between the thickness t at resonance and the refractive index n can be calculated (like in Figure 2f), which means the derivatives of the curve are constant when the refractive index and incident angle fixed. So, the right part of Eq. S12 can be replaced with a constant:

$$\nabla t \cdot S = C \tag{S13}$$

Here, C is a constant that is linked by the refractive index and incident angle. As shown in Eq. S13, we can reduce the thickness gradient, which means that the difference in differential guided-mode resonance is very small to achieve ultra-high sensitivity. We can select a certain condition to determine the order of magnitude of constant C and sensitivity S. According to the measured refractive index of SiO_2 (Figure S16), we can also calculate the relationship curve between the thickness t at resonance and the refractive index n. As shown in Figure S17, from this curve (at fixed incident angle 61.685°), we can obtain the derivative value (at $n = 1.33$) is nearly 3333.3 nm/RIU. Regarding the scale coefficient C_1, we assume that the whole sensing chip precisely maps to the entire CMOS imager region, which is about 80 pixel/mm. Therefore, the constant C is estimated to be 530000 (pixel/RIU) · (nm/mm). As we can see from Figure S17, the curve becomes more intense as the refractive index increases, which means the constant C becomes larger at a higher refractive index. For example, when n is 1.3342, the constant C is nearly 1590000 (pixel/RIU) · (nm/mm).

Additionally, to present the sensitivity independently of the detector's pixel density (CMOS camera with 1608 × 1104 pixels), we can convert the pixel-shift value to an absolute length-shift value. Given that each pixel corresponds to approximately 5 μm, the sensitivity S (990,000 pixels/RIU) can be recalculated as 4,950,000 μm/RIU.

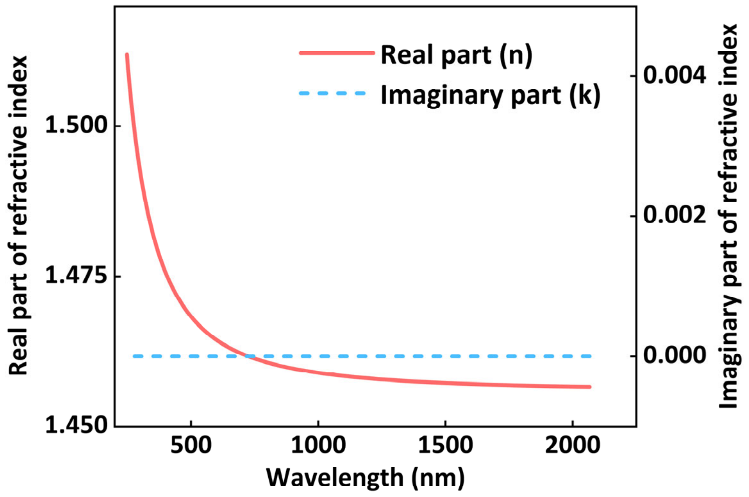

Figure S16. The measured refractive index of SiO_2, including the real part (red solid line) and imaginary part (blue dashed line) of the refractive index. The real part *n* is 1.4626 at 671 nm.

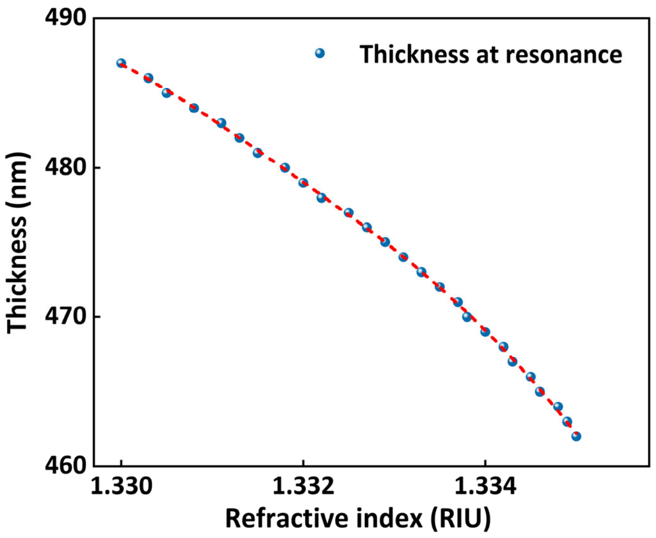

Figure S17. The relationship curve between the thickness at resonance and the refractive index at a fixed incident angle.

11.2 The range-gradient relationship

Here we discuss the relationship between the dynamic range (in one range cycle) and the thickness gradient, as depicted in Figure 4d. The dynamic range in one range cycle under the fixed incident angle condition is determined by the pixel shift range, that is, corresponding to the size of the sensing chip. As the chip size is fixed to be *L*, there exists the relationship below:

$$\int_0^{\frac{L}{2}} \nabla t \cdot dx = t_2 - t_1 \tag{S14}$$

Here, $\nabla t = dt/dx$ (as mentioned in Eq. S11), t_2 and t_1 are the thickness at the position of x=L/2 and x=0, respectively. According to the relationship curve between the thickness t at resonance and the refractive index n, there exists:

$$t_2 - t_1 = t(n_2) - t(n_1) = \int_{n_2}^{n_1} \left(-\frac{dt(n)}{dn}\right) dn \tag{S15}$$

So, there exists:

$$\int_0^{\frac{L}{2}} \nabla t \cdot dx = \int_{n_2}^{n_1} \left(-\frac{dt(n)}{dn}\right) dn \tag{S16}$$

As shown in Figure S17, if we assume that the curve is linear in the small range of n. Then we can obtain:

$$\int_0^{\frac{L}{2}} \nabla t \cdot dx = C_n \cdot \Delta n \tag{S17}$$

Δn is equal to n_2-n_1. C_n is a constant. Eq. S17 describes the relationship between the thickness gradient and dynamic range of refractive index in one range cycle. If we assume that the thickness gradient is a constant, which means the linear gradient in the chip scale, then we can obtain:

$$\Delta n = \frac{L}{2C_n} \nabla t \tag{S18}$$

As we can see from Eq. S18, there exists a linear relationship between the dynamic range and the thickness gradient under the assumptions above. $L/2C_n$ is calculated to be about 0.003 mm/nm. From Eq. S18, we can see that increasing the chip size can also expand the refractive index detection range. However, in many applications the sensing area (i.e., the size of the sensing chip) is fixed or limited, we would prefer to tune the incident angle as a convenient way to extend the refractive index range, facilitating system miniaturization and integration. However, adjusting the incident angle introduces potential measurement errors, particularly during the transition to the next measurement cycle when the angle is reset to return the resonance fringes or rings to their initial position. To mitigate such errors, a possible correction method could involve marking the initial and final positions on the chip. When the camera detects that the resonance rings have reached the final position, an automated control algorithm

could adaptively adjust the incident angle to the next measurement cycle, thereby reducing errors to some extent.

11.3 The refractive index resolution

Here we consider the refractive index resolution, which we also call the detection limit (see Δn in Figure 4c). To distinguish it from the dynamic range above, Δn_L is the refractive index resolution here. There are two methods to calculate the refractive index resolution.

The first one is based on the measurement noise of the CMOS camera for the gray value. As depicted in Figure 4c, only the gray value change resulting from the stripe shift is higher than the measurement noise (or three times the measurement noise) of the CMOS camera, the stripe shift can be affirmative. Thus, we can describe it as:

$$\Delta g = \frac{dg}{dx} S \Delta n_L \geq 3\sigma \tag{S19}$$

Here, Δg is the gray value change resulting from the stripe shift. dg/dx is the derivative of the gray value at the pixel axis (x), which is the stripe fineness. S is the sensitivity of the stripe shift for the refractive index (n). σ is the measurement noise of CMOS camera. Thus, we can obtain

$$\Delta n_L \geq \frac{3\sigma}{S \cdot dg/dx} \tag{S20}$$

As shown in Figure S18, the measurement noise of CMOS σ is about 0.52 (a.u.). Sensitivity S can be assumed as 990000 pixel/RIU. dg/dx is about 1.6 a.u./pixel. According to Eq. S20, the refractive index resolution can be calculated to be 9.85×10^{-7} RIU. If we consider that the gray value change resulting from the stripe shift is higher than the measurement noise, the refractive index resolution can be calculated to be as Δn_L=3.28×10^{-7} RIU.

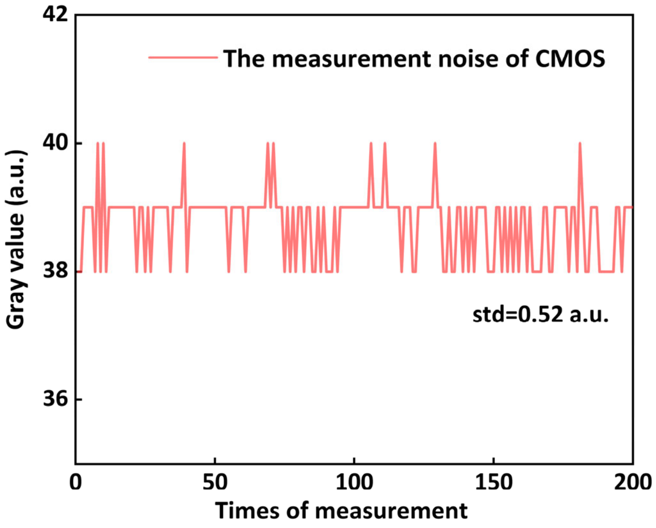

Figure S18. The measurement noise of CMOS near the resonant stripe area. The total number of measurements is 200 times. The standard derivation std, that is σ, is about 0.52 (a.u.).

The second method to calculate the refractive index resolution is directly based on the pixel shift noise under continuous monitoring. It can be described as:

$$\Delta n_L \geq \frac{\sigma_{\text{pixel}}}{S} \quad (S21)$$

As shown in Figure S19, the measurement noise of pixel shift of the stripe σ_{pixel} is about 0.31 pixel. Thus, the refractive index resolution can be calculated to be about Δn_L=3.13×10^{-7} RIU. The refractive index resolution is calculated to be 1.01×10^{-6} RIU, if we consider the measurement noise of pixel shift as 1 pixel (instead of 0.31 pixel).

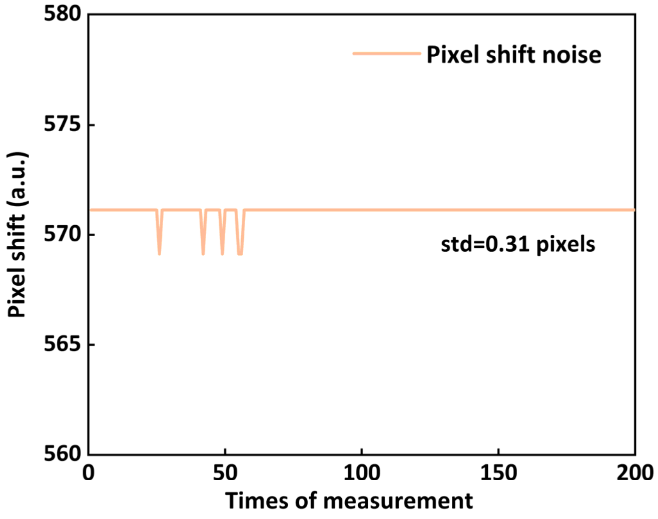

Figure S19. The measurement noise of pixel shift of the stripe. The total number of measurements is 200 times. The std (standard derivation), that is σ_{pixel}, is about 0.31 pixels.

12. The fineness of the stripe and the calculation of the Figure of Merit (FOM)

Based on the measured half width of the stripe, as shown in Figure S20, we can obtain the value of the FOM, referring to the generalized definition of FOM in the spectrum[7,8].

The definition of FOM is shown below:

$$\mathrm{FOM} \geq \frac{S}{\mathrm{FWHM_{stripe}}} \qquad (S22)$$

Here, $\mathrm{FWHM_{stripe}}$ is the full width at half maxima of the stripe. S is the sensitivity. As shown in Figure S20, the minimum value of the stripe FWHM is 57 pixels, and the maximum value is 108 pixels. So, through calculating according to the Eq. S22, the maximum of the FOM can reach 1.7×10^4 $\mathrm{RIU^{-1}}$. Such a high FOM results from the combination of the small gradient of thickness (which brings the ultrahigh sensitivity) and the high Q-factor guided-mode resonance (which brings the high stripe fineness).

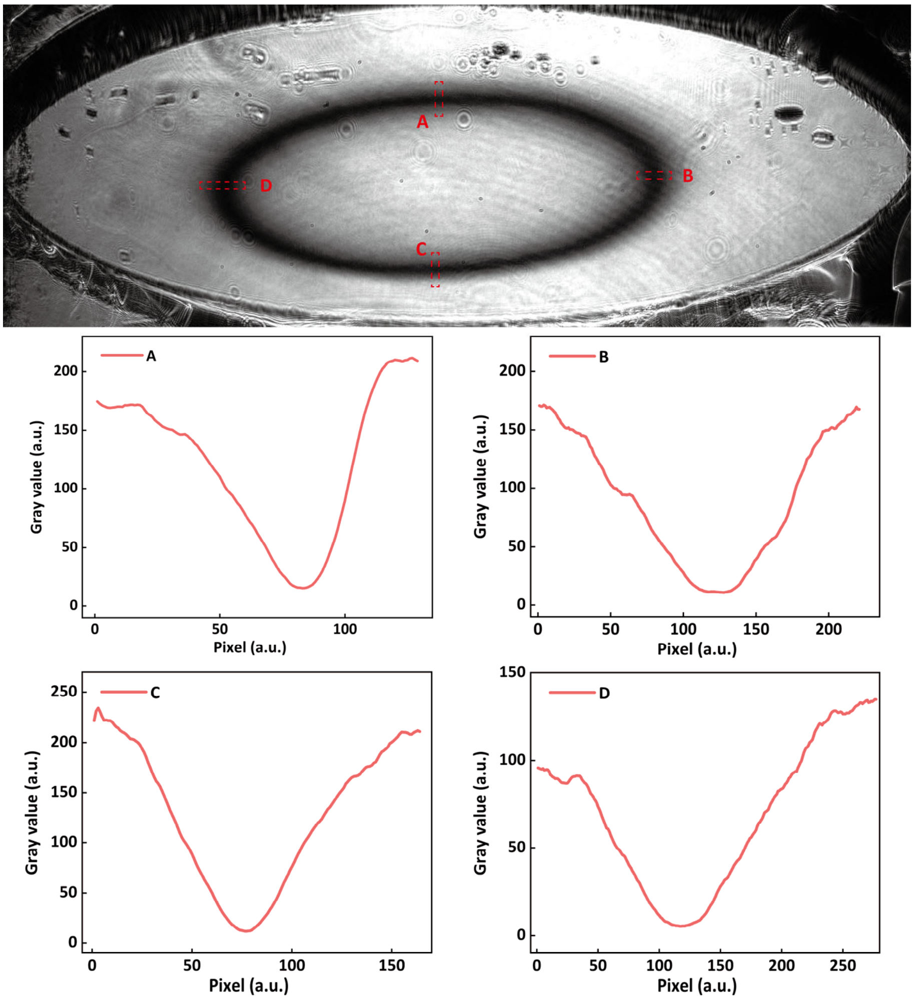

Figure S20. The fineness of the stripe and the calculation of the Figure of Merit (FOM). A, B, C and D in the figure mean the different regions of the resonant stripe, respectively.

13. The repeated measurement error

In Figure 4, we consider the measurement error for the repeated measurement. For example, the experiment was repeated five times for the glucose solution with 0.0% and 0.1% concentration, as shown in Figure S21. The pixel in this figure means the absolute pixel position, instead of the pixel shift in Figure 4. The repeated measurement error is about 0.45 pixel (0.0% concentration) and 0.55 pixel (0.1% concentration).

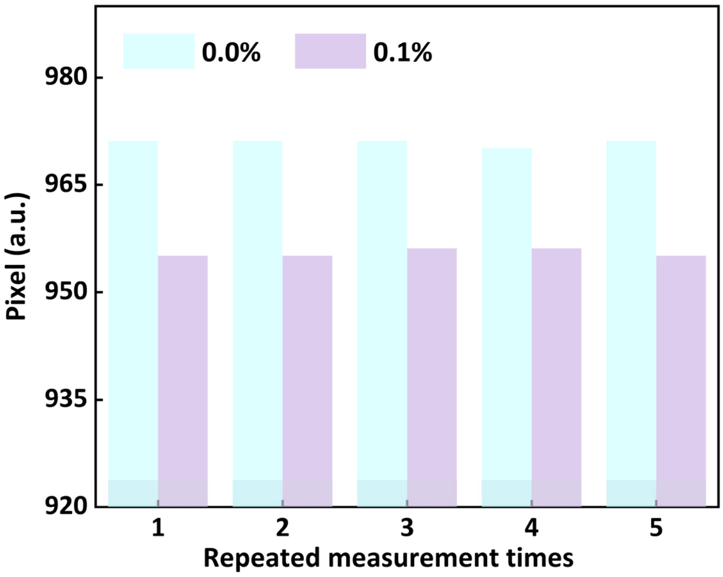

Figure S21. The measurement error of five repeated times for the glucose solution with 0.0% and 0.1% concentration.

14. The method to determine the pixel shift of the ring stripe

We measured the pixel shift based on the diameter of the ring stripe in the horizontal direction (x-direction in the Figure S22), as indicated by the red arrow, rather than averaging over the entire ring. This choice was made because, in prism-coupled imaging, image compression occurs along the direction parallel to light propagation (y-axis in the figure), leading to an elliptical appearance of the ring. Thus, the diameter of the ring in the horizontal direction can reflect the real pixel shift.

To determine the pixel shift, we first performed grayscale analysis on the image to obtain the grayscale distribution along the x-direction. We then identified the pixel position corresponding to the lowest grayscale value as the starting or ending point, which allowed us to accurately calculate the pixel shift.

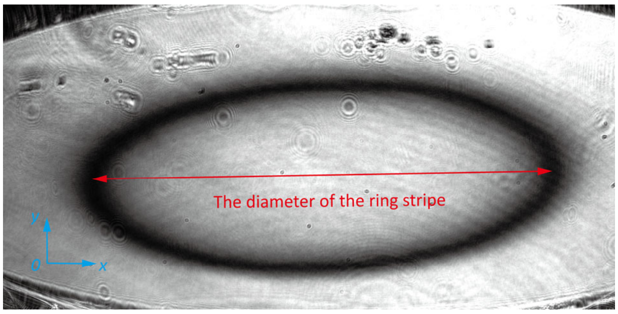

Figure S22. The schematic diagram of pixel shift determination.

15. The detailed modification and some results regarding the biotin test in Figure 5a

The surface modification process involved: Firstly, conducting silanization treatment to the deposited SiO_2 using 2% (v/v) APTES (3-aminopropyltriethoxysilane, $C_9H_{23}NO_3Si$) for 40 min. Secondly, conducting glutaraldehyde (5% (v/v)) treatment for 1 hour. Thirdly, conducting streptavidin incubation (200 μg/mL) for 1 hour. Then blocking other binding sites with 5 mg/mL BSA (bovine serum albumin) for 1 hour. Finally, monitoring the biotin solution (1 nmol/L) binding for 50 min.

We conducted real-time monitoring of the 1 nmol/L biotin binding process, with the resulting resonance stripe shift shown below (Figure S23). The pixel shift means the relative shift pixels compared to the initial process. The stripe moves fast to left from the beginning, that means the increasing refractive index of the surface. Then the stripe moves gradually as the time increasing.

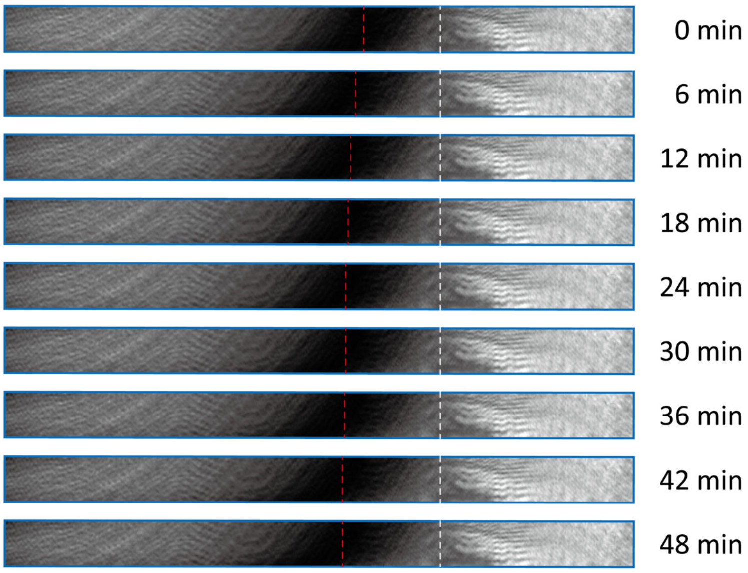

Figure S23. Real-time monitoring of the 1 nmol/L biotin binding process. The red dashed line represents the movement of the stripes, while the white dashed line represents the background that does not move for reference.

As shown in Figure S24, the stripe curve of image gray shows a clear shift as the time increase during the binding process.

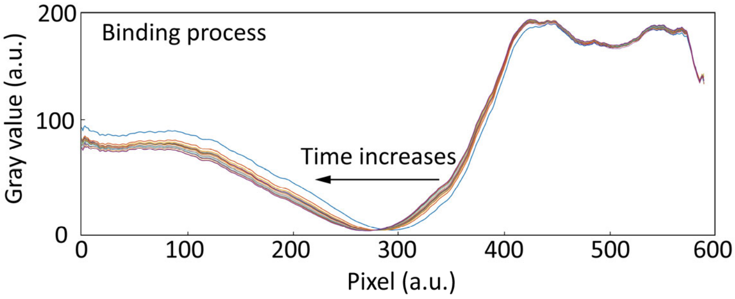

Figure S24. The stripe curve of image gray during the binding process. The arrow in the figure means the direction of time increasing, following with the peak shift.

The pixel shift curve for 1 nmol/L biotin is presented in the Figure S25. A significant shift of over 20 pixels was observed at this low concentration. The binding signal increased rapidly at the initial stage and gradually reached saturation over time.

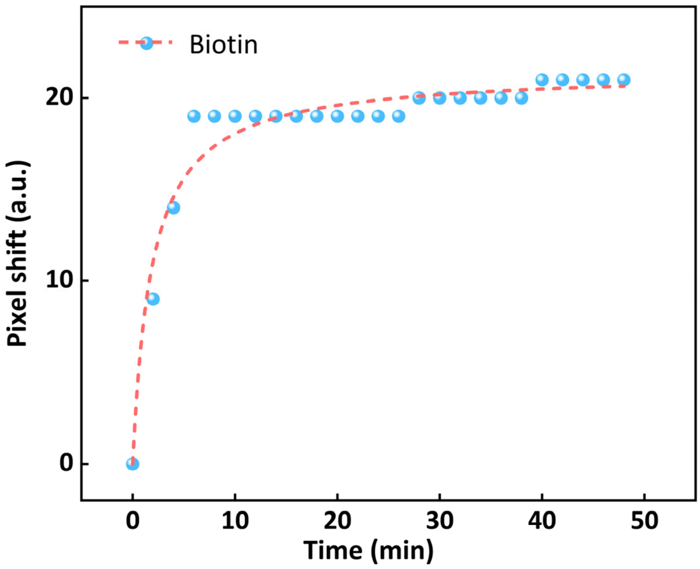

s

Figure S25. The pixel shift curve for 1 nmol/L biotin binding process.

16. The two-dimensional dynamic perception for refractive index or particle distribution

We have already shown the dynamic sensing for the refractive index *n*, which means the information of *n* (*t*) can be measured through our method. To measure the two-dimensional (2D) distribution of *n* near the surface, we need to carry out the angle scanning process. Here, we consider the 2D static distribution of *n* (time-independent), which means *n* (*x*, *y*). Like in the previous discussion, we also make the smooth range assumption for the convenience of analysis.

We first consider the uniform distribution of *n*. As shown in Figure S26, if *n* is uniform, the ring stripe will gradually and uniformly scan every point on the surface, followed by the incident angle scanning. The uniform *n* can be calculated through the *n*-*θ* curve (like the *n*-*θ* curve in Figure 2) of any point on the surface.

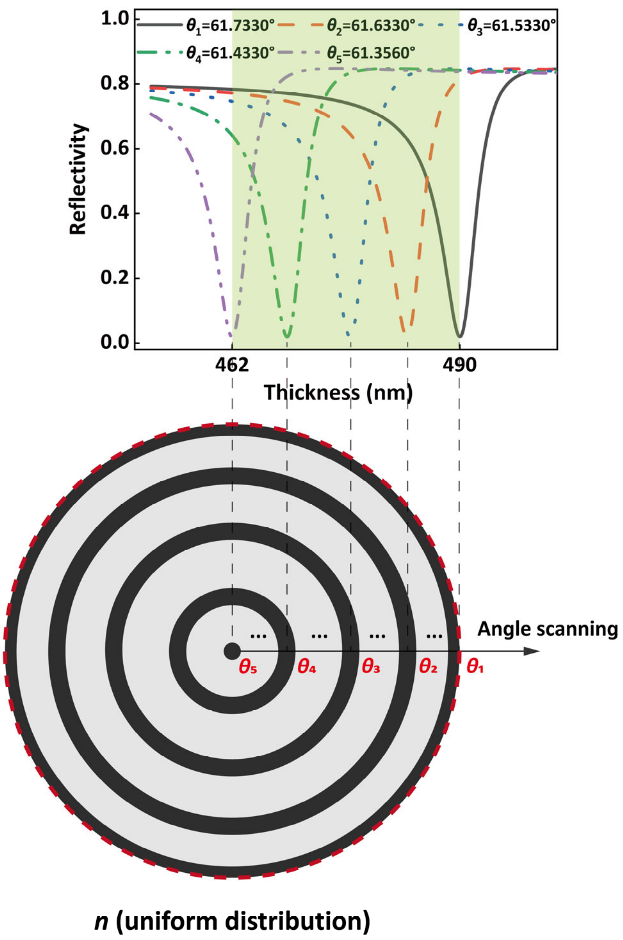

Figure S26. The angle scanning for the special case of uniform distribution of *n*.

Furthermore, we consider that the distribution of *n* is nonuniform, as shown in Figure S27a. As a simple special for illustration, we assume that there are three areas with different refractive

indices, namely, Area A (n_1) and Area B (n_2) in the otherwise uniform background Bg. (n_0). We also carry out the incident angle scanning process. Owing to the different n of those areas (causing different resonance angles), the scanned stripes will exhibit a misalignment phenomenon (as compared with the stripes in the uniform background), as shown in Area A and Area B in Figure S27a. The calculation of n_0, n_1 and n_2 is shown in Figure S27b. As we carry out the angle scanning to obtain the resonant angle of some point in area Bg., area A and area B, respectively, we can calculate the corresponding n according to the n-θ curve of this point, as shown in Figure S27b. Each point with a certain known thickness has a certain n-θ curve. Therefore, if we measure the resonant angle of each point on the surface, we can calculate the refractive index of every point, which is n (x, y). As we can know from Figure S26, the scanning range of angles is about 0.38°. Thus, this angle scanning can be completed very fast, which means the measurement is nearly real-time. Therefore, the two-dimensional time-domain distribution of refractive index can be measured and calculated based on our method. If we have known the relationship between the concentration of certain particles and refractive index, the two-dimensional time-domain distribution of certain particles can also be obtained.

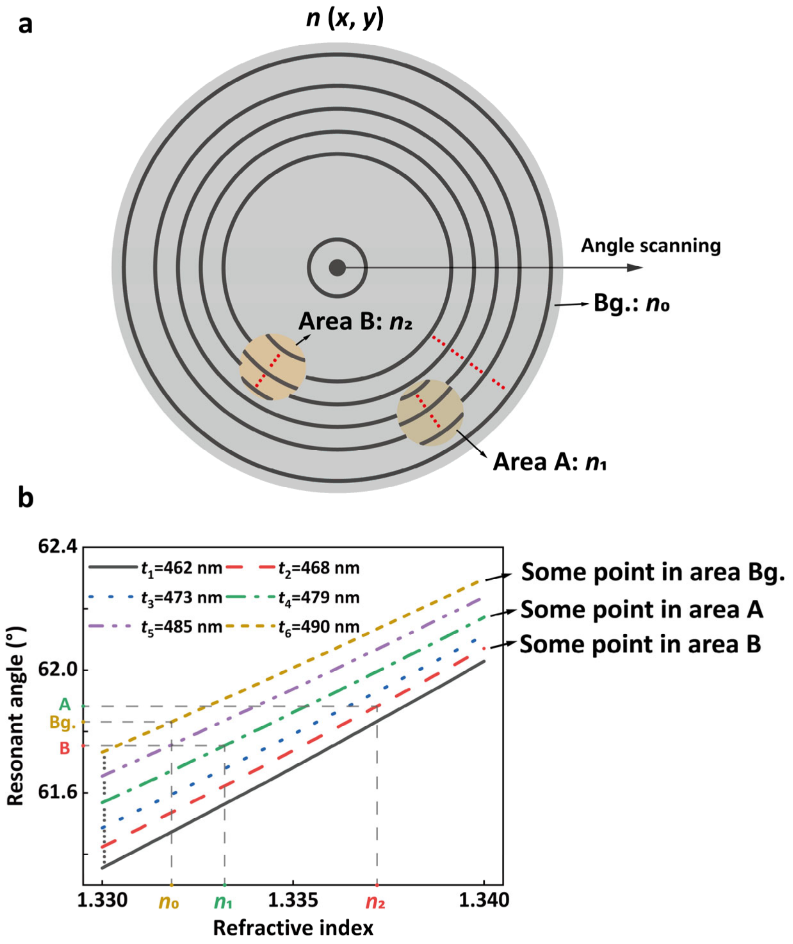

Figure S27. The angle scanning and calculation for the uneven distribution of n. **(a)** The angle scanning process for the uneven n, including the background area Bg., area A and area B on the surface. As a simple special case for illustration, we assume the refractive index in each area is uniform: area Bg. (n_0), area A (n_1) and area B (n_2). **(b)** The n-θ curve for different thicknesses. Each point holding a certain thickness, has a certain n-θ curve.

17. The details on the sensitivity of the imaging sensor to variations in the incident angle and the collimation of the incident waves

As shown in Figure 1b, the sensitivity S is defined as:

$$S = \frac{2|R_B - R_A|}{|n_B - n_A|} \tag{S23}$$

It can also be expressed as:

$$S = \frac{2\Delta R}{\Delta n} \tag{S24}$$

In Figure 2e, the relationship between the refractive index n and the thickness t at resonance is given as:

$$n = f(t) \tag{S25}$$

Using Eq. S25, Eq. S24 can be reformulated as:

$$S = \frac{2\Delta R}{\Delta f(t)} = \frac{2\Delta R}{\Delta t}\frac{1}{f'(t)} = C\frac{1}{f'(t)} \tag{S26}$$

Here, C is a constant determined by the fixed structure of the sensing chip. The sensitivity S is inversely proportional to the $f'(t)$. Moreover, the influence of the incident angle is reflected in $f'(t)$.

In Figure 2g, the relationship curve is plotted under a fixed incident angle. Through further calculations, we obtained relationship curves of $1/f'(t)$ for different incident angles. As shown in Figure S28, $1/f'(t)$ values (the sensitivity S is inversely proportional to the $f'(t)$) remain nearly identical under different incident angles in the low refractive index region, while in the high refractive index region, sensitivity increases as the incident angle decreases.

Regarding incident wave collimation, it broadens the resonance fringes, with sensitivity determined by the average of the maximum and minimum incident angles. In the low refractive index region, the collimation has minimal impact on sensitivity, whereas in the high refractive index region, it enhances sensitivity while simultaneously widening the resonance fringes.

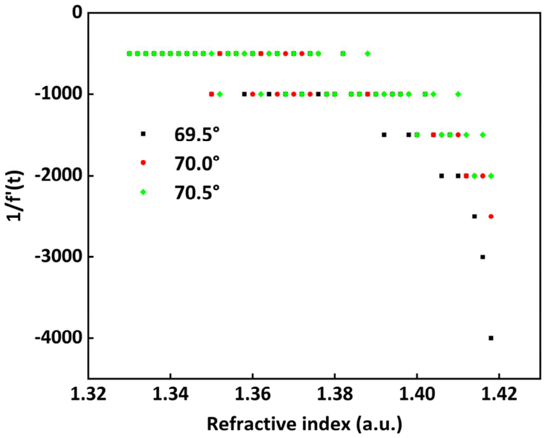

Figure S28. The relationship between 1/*f*'(*t*) and the refractive index under different incident angles.

18. The comparison between image-based refractive index sensors and other types of refractive index sensors

Table S1. The comparison between image-based sensors and other types of ones.

Type		Sensitivity	LOD	Preparation complexity	System complexity
Spectrum shifting/splitting[9,10]		Low	High	Medium	Medium
Phase changes[11,12]		High	Low	Medium	High
Amplitude changes[13,14]		Medium	Medium	Medium	Low
Image-based sensors	Other types[15,16]	Low (10^3 pixel/RIU)	High	High	Low
	**Our work**	**High (10^6 pixel/RIU)**	**Low**	**Low**	**Low**

Notes: LOD means limit of detection.

19. Detailed explanation of dGMR and the physical mechanism behind high sensitivity

dGMR refers to differential guided-mode resonance, where the critical component is the differential mechanism. As shown in the schematic diagram of Figure 1b, we construct two guided-mode resonances with slight spatial differences in thickness to form a sensing unit. This enables the detection of refractive index changes through spatial shifts of resonance fringes. Based on the dGMR sensing mechanism and theoretical framework, we derive the sensitivity expression. Sensitivity depends on the resonance structure's intrinsic sensing performance and the spatial thickness difference (or gradient). As shown in Figure 4a, the smaller spatial thickness gradient greatly enhances the sensing sensitivity.

Previous works[15,16] involve the construction of geometric metasurfaces. Analyzing their systems under the dGMR framework reveals that their resonance structures have insufficient sensing performance, and their spatial geometric gradients are too large. As a result, their sensitivity is three orders of magnitude lower than that of the proposed dGMR sensor.

20. The repeatability assessment for the sensor chip fabrication based on PECVD

To evaluate the repeatability of our PECVD-based fabrication process, we prepared five sensor chips from the same batch under identical deposition conditions, maintaining fixed gas flow rates, deposition power, chamber pressure, and a consistent deposition duration of 2 minutes and 30 seconds, as shown in Figure S29.

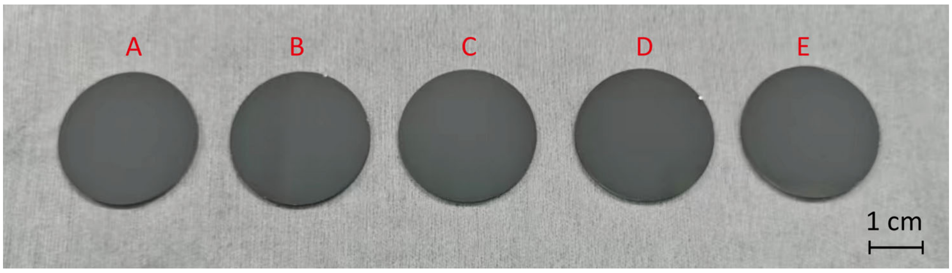

Figure S29. Five gradient sensor chips fabricated via PECVD from the same batch. From left to right, they are labeled as Sample A to Sample E. The scale bar represents 1 cm.

As demonstrated in Figure 4d and detailed in Supporting Information (Section 11.1), the sensitivity of the sensor chip, under identical incident angle and refractive index conditions, is primarily determined by the engineered thickness gradient. Thus, verifying the reproducibility of the PECVD deposition process directly translates to assessing the uniformity and consistency of the thickness gradient across multiple fabrication samples, which also means the repeatability of the thickness fabrication based on PECVD deposition.

To further confirm the repeatability of samples from the same batch, we reconstructed the thickness profile using angle-resolved measurements, following the approach described in Figures 3d and 4a. Five sensor chips were analyzed using angular scanning, and the corresponding imaging results at the same incident angle are shown in Figure S30. All five chips exhibit identical resonance ring features, providing preliminary validation of the reproducibility of thickness-gradient sensors fabricated via PECVD.

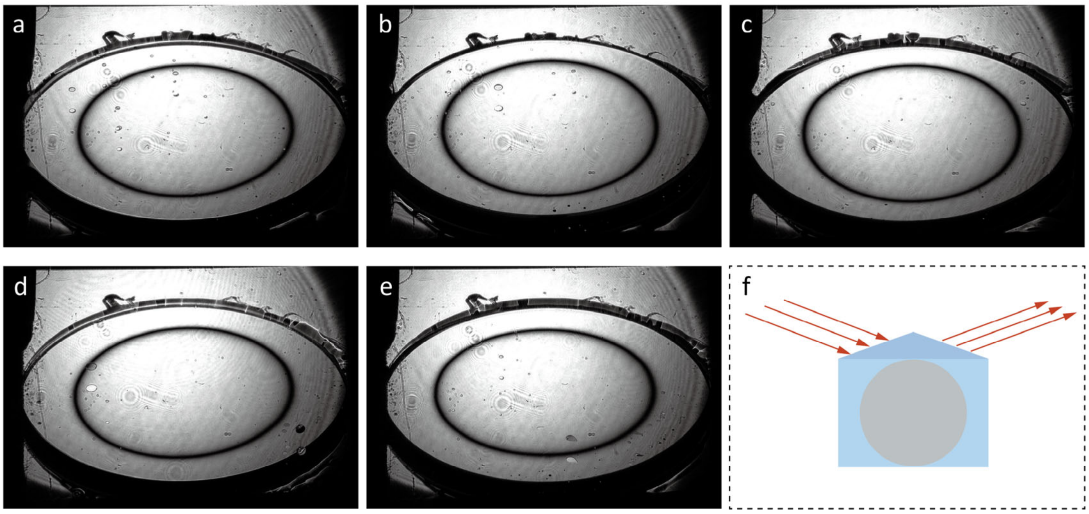

Figure S30. The imaged resonance ring-stripe of five sensor chips from the same batch under the same incident angle. (a)-(e) is corresponding to the sample A-E. (f). The schematic diagram of the partial test system.

Figure S31 presents the reconstructed thickness profiles along a horizontal cross-section, revealing high consistency among the five samples. At the middle area (point P) of Figure S31, the fabrication standard deviation among the five chips is 2.4%, indicating a good reproducibility in our PECVD-based fabrication method.

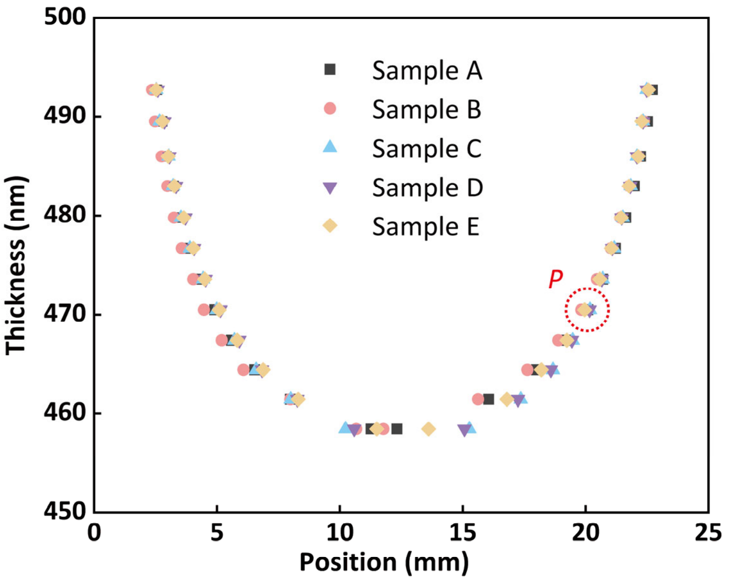

Figure S31. The reconstructed thickness profiles of five samples from the same batch.

21. The detailed fabrication process for the sensor chip

In Figure 3, we controlled the SU-8 thickness by adjusting the exposure dose, with the fabrication process outlined in Figure S32.

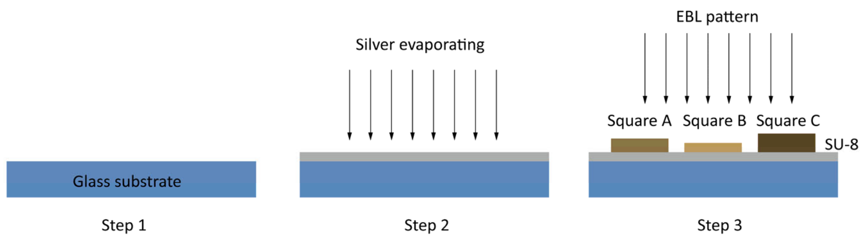

Figure S32. The fabrication of sensor chip based on SU-8 exposure dose controlling in Figure 3. Squares A, B and C means the square film with different exposure dose.

For the sensor chip in Figure 4, we leveraged the inherent deposition variations of PECVD to create a continuous thickness gradient, enabling a lithography-free approach for fabricating natural thickness variations. The detailed fabrication process is shown in Figure S33.

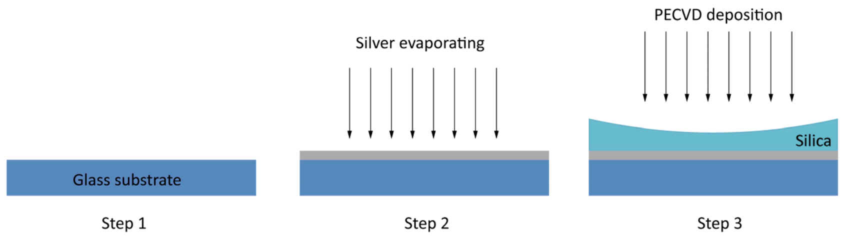

Figure S33. The fabrication of sensor chip based on PECVD deposition in Figure 4.

22. The thickness controlling experiment through EBL dose-modulation patterning

We fabricated SU-8 samples at lower exposure doses of 2.0, 2.5, 3.0, and 3.5 μC/cm^2, with four samples per dose to evaluate thickness variations. As shown in Figure S34, the SU-8 thickness initially increases rapidly with dose and then gradually slows as the dose continues to rise. The thickness deviation among samples with the same dose is less than 3 nm, indicating that precise thickness control can be achieved by adjusting the exposure dose, enabling the fabrication of subtle thickness variations and gradients.

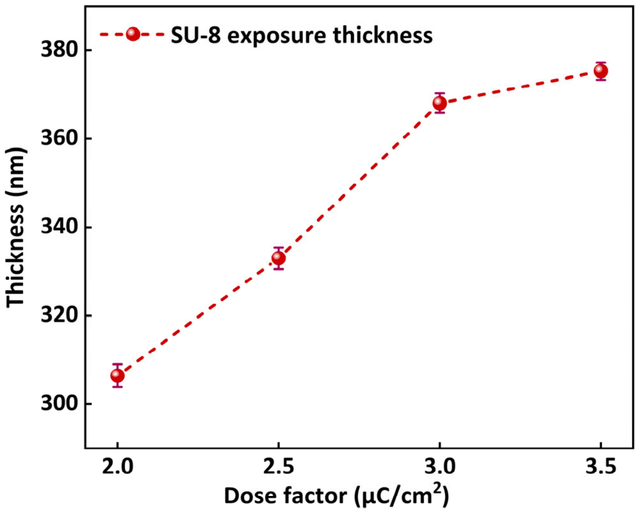

Figure S34. The relationship curve between the SU-8 films thickness and the exposure dose.

References

1 Yao, J. *et al.* Plasmonic anapole metamaterial for refractive index sensing. *Photonix* **3**, 23 (2022).

2 Hsiao, H. H., Hsu, Y. C., Liu, A. Y., Hsieh, J. C. & Lin, Y. H. Ultrasensitive Refractive Index Sensing Based on the Quasi-Bound States in the Continuum of All-Dielectric Metasurfaces. *Advanced Optical Materials* **10**, 2200812 (2022).

3 Guo, T. B., Evans, J., Wang, N. & He, S. L. Monolithic chip-scale structural color filters fabricated with simple UV lithography. *Opt. Express* **27**, 21646-21651 (2019).

4 Yeh, Y. L. Real-time measurement of glucose concentration and average refractive index using a laser interferometer. *Opt. Lasers Eng.* **46**, 666-670 (2008).

5 Giorgis, F., Descrovi, E., Summonte, C., Dominici, L. & Michelotti, F. Experimental determination of the sensitivity of Bloch Surface Waves based sensors. *Opt. Express* **18**, 8087-8093 (2010).

6 Kedenburg, S., Vieweg, M., Gissibl, T. & Giessen, H. Linear refractive index and absorption measurements of nonlinear optical liquids in the visible and near-infrared spectral region. *Opt. Mater. Express* **2**, 1588-1611 (2012).

7 Slaby, J. & Homola, J. Performance of label-free optical biosensors: What is figure of merit (not) telling us? *Biosens. Bioelectron.* **212**, 114426 (2022).

8 Wang, Z. L., Wang, X. & Wang, J. L. Research Advance on the Sensing Characteristics of Refractive Index Sensors Based on Electromagnetic Metamaterials. *Adv. Condens. Matter Phys.* **2021**, 2301222 (2021).

9 Shen, Y. *et al.* Plasmonic gold mushroom arrays with refractive index sensing figures of merit approaching the theoretical limit. *Nat. Commun.* **4**, 2381 (2013).

10 Park, J. H. *et al.* Symmetry-breaking-induced plasmonic exceptional points and nanoscale sensing. *Nature Physics* **16**, 462-+ (2020).

11 Song, M. T. *et al.* Enhanced on-chip phase measurement by inverse weak value amplification. *Nat. Commun.* **12**, 6247 (2021).

12 Sahoo, P. K., Sarkar, S. & Joseph, J. High sensitivity guided-mode-resonance optical sensor employing phase detection. *Scientific Reports* **7**, 7607 (2017).

13 Haider, F., Aoni, R. A., Ahmed, R. & Miroshnichenko, A. E. Highly amplitude-sensitive photonic-crystal-fiber-based plasmonic sensor. *Journal of the Optical Society of America B-Optical Physics* **35**, 2816-2821 (2018).

14 Monfared, Y. E., Hajati, M., Liang, C. H., Yang, S. C. & Qasymeh, M. Quasi-D-Shaped Fiber Optic Plasmonic Biosensor for High-Index Analyte Detection. *Ieee Sensors Journal* **21**, 17-23 (2021).

15 Li, G. H. *et al.* Cost-Effective Nanophotonic Metasurfaces with Spatially Gradient Structures for Ultrasensitive Imaging-Based Refractometric Sensing. *Small Methods* **8**, 2300873 (2024).

16 Min, S. Y. *et al.* Ultrasensitive Molecular Detection by Imaging of Centimeter-Scale Metasurfaces with a Deterministic Gradient Geometry. *Adv. Mater.* **33**, 2100270 (2021).
